# Antibiotic Use and Misuse during Pregnancy and Delivery: Benefits and Risks

**DOI:** 10.3390/ijerph110807993

**Published:** 2014-08-07

**Authors:** Begoña Martinez de Tejada

**Affiliations:** 1 Department of Obstetrics and Gynecology, Geneva University Hospitals, 1205 Geneva, Switzerland; 2 Faculty of Medicine, University of Geneva, 1205 Geneva, Switzerland; E-Mail: begona.martinezdetejada@hcuge.ch; Tel.: +41-22-382-6816; Fax: +41-22-382-4146

**Keywords:** antibiotics, pregnancy, risk, fetal, neonatal, infant, morbidity

## Abstract

Although pregnancy is considered as a physiological state, most pregnant women in developed countries receive multiple medications to prevent maternal or neonatal complications, with antibiotics among the most frequently prescribed. During pregnancy, antibiotics are often prescribed in the context of preterm labor, intrapartum fever, prevention of neonatal Group B *Streptococcus* fever, and cesarean section. Outside this period, they are commonly prescribed in the community setting for respiratory, urinary, and ear, nose and throat infection symptoms. Whereas some of the current indications have insightful reasons to justify their use, potential risks related to overuse and misuse may surpass the benefits. Of note, the recent 2014 World Health Assembly expressed serious concern regarding antibiotic resistance due to antibiotic overuse and misuse and urged immediate action to combat antibiotic resistance on a global scale. Most studies in the obstetrics field have focused on the benefits of antibiotics for short-term maternal and neonatal complications, but with very little (if any) interest in long-term consequences.

## 1. Introduction

Most pregnant women are exposed to some type of medication during pregnancy. Drugs prescribed during pregnancy can exercise a teratogenic effect on fetuses, and those prescribed during breastfeeding can also impact on infant health. Antibiotics are among the more frequently prescribed types of medication during pregnancy and lactation [1]. Current estimates suggest that >40% of pregnant women are given some type of antibiotic immediately prior to delivery [2], either for prevention of neonatal Group B *Streptococcus* (GBS) sepsis or cesarean prophylaxis [3,4]. If other antibiotic indications during pregnancy are taken into account, such as the screening and treatment of asymptomatic bacteriuria or bacterial vaginosis (BV), as well as the overuse of antibiotics to treat respiratory and genital infections, it is evident that the huge majority of fetuses today have been exposed to antibiotics before delivery. Whereas some of these therapeutic strategies have been shown to be beneficial to reduce short-term maternal and neonatal complications, their long-term effects are by far less well known. We review here some of the most common indications for prescribing antibiotics during pregnancy and discuss the potential associated risks, including strategies aimed at reducing these risks. When prescribing antibiotics to a pregnant woman, we should ask ourselves: are they really indicated and are we doing more harm than good?

## 2. Obstetrical Antibiotic Indications during Pregnancy

### 2.1. Prevention of Preterm Birth

The World Health Organization defines preterm delivery or birth as one that occurs at more than 20 weeks’ gestation, but less than 37 weeks [5]. Preterm birth is the single largest contributor to poor outcome in the perinatal period. In developed countries, the incidence of preterm delivery varies between 7% and 11% [6,7]. Despite advances in obstetrical care, the risk of prematurity has not decreased over the past 40 years [8]. Prematurity remains a leading cause of neonatal morbidity and mortality in developed countries, accounting for 60%–80% of deaths of infants without congenital anomalies. Although births at less than 32 weeks’ gestation represent only 1%–2% of all births, they are responsible for 60% of perinatal mortality and 50% of long-term neurological morbidity [5,9]. The economic consequences are very important as one-third of expenses due to neonatal care and one-tenth of expenses due to general pediatric care are caused by premature births. As an example, the neonatal care costs of an infant with a birth weight of 500–700 g in the USA is estimated at approximately USD $225,000 [10,11].

During the last 20 years, increasing research has focused on identifying causal etiologies of preterm birth. A large number of associated risk factors have been identified [12]. Among these, maternal causes of infectious origin are the most frequently found [13,14,15]. The potential causal link between infection and preterm birth is consistent with models suggesting that preterm labor is triggered by an inflammatory response mediated by pro-inflammatory cytokines such as interleukin (IL)-1beta, IL-6, IL-8, and tumor necrosis factor (TNF)-alpha [16,17,18,19,20,21,22].

Laboratory and clinical data have shown a link between spontaneous preterm labor and delivery and both systemic and ascending genital tract infections [13,23]. Both clinical and subclinical chorioamnionitis are much more common in preterm than term deliveries and may account for 50% of preterm births before 30 weeks’ gestation [14]. It seemed therefore logical that antibiotics might have a positive role in the treatment of preterm labor. However, antibiotic treatment in the context of preterm labor with intact membranes has been shown ineffective in preventing either preterm birth or neonatal morbi-mortality [24,25]. A recent Cochrane review including 14 studies randomizing a total of 7837 women with preterm labor and intact membranes showed no significant difference in perinatal or infant mortality, in preterm birth, or in any other clinically important short-term outcomes for the infant. Maternal infection was reduced with the use of any prophylactic antibiotics compared with placebo (average risk ratio (RR): 0.74; 95% confidence interval (CI) = 0.63–0.86; number needed to treat to benefit (NNTB): 34; 95% CI = 24–63) and with any beta-lactam antibiotic compared with non-beta-lactams (RR: 0.80; 95% CI = 0.69–0.92; NNTB: 47; 95% CI = 31–119). In addition, compared with non-beta-lactam antibiotics, any beta-lactam was associated with an increase in maternal adverse drug reaction (RR 1.61, 95% CI = 1.02–2.54; number needed to treat to harm (NNTH): 17; 95% CI = 7–526) [26].

By contrast, antibiotics have been shown to be beneficial in preventing maternal and short-term neonatal complications in the context of premature rupture of membranes (PROM) before term [27,28]. A review including 22 trials, involving 6872 women and babies, showed that the use of antibiotics following PROM was associated with statistically significant reductions in chorioamnionitis (RR: 0.66; 95% CI = 0.46–0.96) and a reduction in the numbers of babies born within 48 h (average RR: 0.71; 95% CI = 0.58–0.87) and 7 days of randomization (average RR: 0.79; 95% CI = 0.71–0.89). The following markers of neonatal morbidity were reduced: neonatal infection (RR: 0.67; 95% CI = 0.52–0.85); use of surfactant (RR: 0.83; 95% CI = 0.72–0.96); oxygen therapy (RR: 0.88; 95% CI = 0.81–0.96); and abnormal cerebral ultrasound scan prior to discharge from hospital (RR: 0.81; 95% CI = 0.68–0.98). On the other hand, antibiotics had very little impact on infants’ health at 7 years of age [26]. Despite a lack of evidence of longer-term benefit in childhood, the advantages related to short-term morbidities are such that antibiotics are recommended to be prescribed in the context of PROM before term.

The diagnosis and treatment of asymptomatic bacteriuria, BV, and gonorrhea appeared to reduce the risk of preterm birth [23,29,30,31]. Nevertheless, recent studies seem to show that the expected benefit might be lower―if any at all [30,32]. As an example, most studies assessing the benefit of screening for BV and subsequent treatment with the objective of preventing preterm birth have been negative [29,32,33,34]. Some authors considered that this lack of effect was due to treatment being initiated too late and with the wrong antibiotic (metronidazole instead of clindamycin) [23,35]. However, a very recent French study including women in early pregnancy treated with clindamycin during 4 days and repeated 1 week later did not show any benefit either [36]. Doubts about the effectiveness of screening and treating BV during pregnancy persist as the regimen used in this latter study is shorter than the recommended one (4 days instead of 7). More studies are needed before recommending screening and treatment of BV during pregnancy and these should include long-term infant outcomes. Regarding other genital infections, although an increased rate of preterm delivery has been described in women with GBS, Chlamydia, *Trichomonas vaginalis*, and syphilis, treatment of these infections is primarily aimed at preventing perinatal transmission or maternal infection [3,30].

There is no doubt that infection and inflammation are clearly related to the risk of preterm birth, but it has not been established whether antibiotic therapy can prevent it. Moreover, antibiotic treatment in the context of prevention of preterm birth might be harmful [24,26,37].

### 2.2. Prevention of GBS Neonatal Disease

GBS is one of the leading causes of neonatal (e.g., early and late sepsis, meningitis) and maternal infectious complications (chorioamnionitis, sepsis, endometritis) [38,39]. GBS early onset disease (EOD) is defined as occurring within the first week after birth and late onset disease as GBS neonatal complications occurring after one week of birth. Maternal intrapartum GBS colonization is the primary risk factor for EOD in infants and approximately 10%–30% of pregnant women are colonized with GBS in the vagina or rectum. It has been shown that intrapartum antibiotic prophylaxis (IAP) for colonized women decreases the risk of vertical transmission of GBS, as well as the risk of EOD [40]. The reference standard for the detection of GBS colonization is considered to be culture in broth-enriched vaginal-rectal swabs. As culture takes at least 48–72 h, it cannot be used for detection of GBS colonization during labor. Therefore, it is recommended to perform GBS culture from a recto-vaginal swab taken at 35–37 weeks’ gestation. Several national organizations, such as the United States Centers for Disease Control and Prevention (CDC) and the Swiss Society of Obstetrics and Gynecology, currently recommend antenatal screening with vaginal-rectal cultures and selective IAP administration to GBS-positive women [3] and it has been reported that adherence to these recommendations has decreased the incidence of GBS EOD (from 1.7 cases per 1000 live births in the early 1990s to 0.34–0.37 cases per 1000 live births in recent years) [3,41,42].

However, several problems exist with these recommendations. In the absence of systematic screening or widespread IAP, the incidence of EOD in some countries (e.g., the United Kingdom (UK)) is much lower (0.5/1000 births) and similar to that observed in the USA after universal screening and IAP, despite comparable vaginal carriage rates [43,44]. Studies have shown a low sensitivity (33%–50%) of antenatal GBS culture to detect colonization during labor [45,46,47,48]. Cases of EOD in infants born at term have occurred among mothers with negative antenatal GBS culture results and it has been reported that at least 10% of this group of women turned positive at labor [49]. Based on current guidelines, these women do not receive IAP and their neonates are at risk of EOD [48]. Of note, neonates born preterm are at highest risk of EOD (risk of 1 in 350–450 depending on gestational age at delivery). As recto-vaginal culture is performed only at 35–37 weeks, their maternal colonization status is not usually known at labor and, therefore, IAP is given systematically―irrespective of colonization status.

The decision to perform systematic GBS antenatal screening is not universally accepted. Some countries, such as the UK, apply mostly a risk factor policy [50,51]. Nevertheless, studies have shown that a very low percentage of newborns with EOD had risk factors (53% in a Finnish study) [52]. Conversely, since colonization with GBS concerns less than 20% of pregnant women, there is a risk of overtreatment when using the risk factor strategy.

When choosing a strategy for the prevention of GBS neonatal sepsis, experts and national societies decide upon the number of women and fetuses they are willing to treat in order to prevent one case of GBS EOD. Using the approach of systematic antenatal screening and IAP to colonized women, between 700 and 1000 women and their fetuses are treated to prevent one case of GBS EOD. With the risk-based strategy, around 1100 women and their fetuses are treated to prevent one case of GBS EOD, many of them without being at risk as the mother is not colonized. Therefore, it is of extreme importance to choose a strategy attempting to expose the highest at-risk population to antibiotic prophylaxis, which is colonized women delivering preterm.

### 2.3. Chorioamnionitis during Labor

Intrapartum clinical chorioamnionitis, usually diagnosed on the basis of the presence of fever, uterine tenderness, maternal or fetal tachycardia, foul-smelling or purulent amniotic fluid, leucocytosis or elevated C-reactive protein (CRP), is present in 4%–10% of women in labor [53]. Chorioamnionitis is associated with a high risk of necrotizing enterocolitis (odds ratio (OR): 1.24; 95% CI = 1.01–1.52), and infant cerebral palsy (OR: 2.42; 95% CI = 1.52–3.84), as well as maternal complications (e.g., endometritis and sepsis) [54,55,56]. Broad-spectrum antibiotics, such as co-amoxiclav plus gentamicin, are usually the standard of care as treatment allows to prevent maternal complications [57,58]. Unfortunately, the benefit of such a treatment for the prevention of infant cerebral palsy is less well established as the risk persists irrespective of antibiotic administration. Whereas there is no doubt that antibiotics should be prescribed in the context of chorioamnionitis, it is also clear that the correct diagnosis should be made before starting treatment.

### 2.4. Prevention of Maternal Infectious Morbidity after Cesarean Section

Cesarean section rates continue to increase and rates in developed countries are as high as 30%–40%. Women delivering by cesarean are at a 5- to 30-fold increased risk of postpartum infection-related complications (including endometritis, sepsis, urinary tract infection. and surgical site infection (1.1%–25%)) compared with those delivering vaginally (0.2%–5%) [59,60]. Women undergoing cesarean delivery (CD) before labor or membrane rupture are at a much lower risk of infection-related complications (5.5%–17.3%) than those having a CD during labor, where the risk of postpartum infection (without antibiotic prophylaxis) is as high as 8%–30% [60].

Antibiotic prophylaxis for women undergoing CD has been proven to be beneficial in decreasing postpartum infectious morbidity both in high-risk (in labor post-membrane rupture) [61] or low-risk patients (non-laboring with intact membranes) [62,63]. In a systematic review of over 80 studies on the use of prophylactic antibiotics for CD, the Cochrane Collaboration specifically examined the effect of prophylactic antibiotics on the rate of maternal postpartum fever, wound infection, endometritis, urinary tract infection, serious infectious morbidity/death, as well as maternal side-effects and length of hospital stay [64]. For all CDs (both elective and emergency), the only outcome that increased following prophylactic antibiotics was maternal side-effects. For all other outcomes, the use of antibiotics was associated with a statistically significant reduction, with an effect size of 40%–65%. Endometritis and wound infection were reduced following both elective and emergency CD.

Until recently, consensus was to give antibiotic prophylaxis after umbilical cord clamping in order to avoid the newborn being exposed to any potential infection. Regardless of this prophylaxis, postpartum infectious morbidity is still high in some countries, such as the USA. Recently, research has been conducted to evaluate whether antibiotic prophylaxis before skin incision (as for gastrointestinal surgery) could further reduce postpartum maternal complications. Several reports have shown that antibiotics given before skin incision were more effective in reducing endometritis than when given after cord clamping (RR: 0.57; 95% CI = 0.36–0.90) [65,66,67]. However, it can be argued that this approach has several problems. (1) Very few studies have investigated neonatal morbidity. These have shown that the rate of short-term complications was similar if antibiotics were given before or after cord clamping (neonatal sepsis (0.82; 95% CI = 0.47–1.42); suspected neonatal sepsis requiring a workup (RR: 0.94; 95% CI = 0.72–1.22); or neonatal intensive care unit admissions (RR: 0.90; 95% CI = 0.62–1.28)) [67]. Of note, there is no prospective study investigating the mid- or long-term effect on the infant. (2) The results are not universal with some studies showing no difference in maternal complications regardless of whether antibiotics are given before skin incision or after cord clamping [4,68,69,70]. (3) The rate of postoperative morbidity in the USA is much higher than in other developed countries―probably due to high rates of obesity and diabetes. Therefore, although antibiotic prophylaxis diminishes the risk of postpartum infectious maternal morbidity after cesarean section, more studies are needed before changing the current standard of care, which is to give them after cord clamping [71].

## 3. Antibiotic Misuse, Risks Associated with Antibiotic Prescription, and Strategies for Risk Reduction

There is a vast amount of data showing that antibiotic use is not free of serious adverse events. Among other complications, antibiotics have been associated with allergic reactions, gastrointestinal disturbances, cardiac arrhythmia, and death [72]. A major problem associated with antibiotic overuse is the development of multiresistant bacteria [73,74]. The necessity to treat most cases of symptomatic bacterial infections is clear, but it is also clear that during the last 20 years there has been an inappropriate overuse of antibiotics to treat respiratory, ear, nose and throat, urinary, or genital infections. It can be stated also that antibiotics have been and still are overused in obstetrics.

The widespread use of antibiotics is associated with maternal anaphylaxis (2.7 cases per 100,000 deliveries) and cases have been described both during pregnancy and the peripartum period (for GBS EOD prevention and during cesarean section) [75,76,77]. Maternal anaphylaxis has a potentially devastating effect on fetal oxygenation. Following maternal hypotension, the fetus compensates for the decreased blood flow by the redistribution of blood to vital organs, increased oxygen uptake and tissue oxygen extraction, and decreased body movements. When these mechanisms fail, the fetus is at risk of hypoxic-ischemic encephalopathy and permanent central nervous system damage. Maternal cardiac arrest is potentially devastating to the fetus and mandates immediate CD. The risk of infant neurological damage appears after just 5 min in addition to the risk of fetal/neonatal death [78,79].

It has been suggested that exposure to antibiotics during fetal/neonatal life affects the development of allergic diseases via their adverse and possible long-term effect on gut microbiota of both the mother and child and vaginal microbiota of the mother. Antibiotic use may delay and interfere with the early colonization of the child’s gut microbiota [80]. In turn, this delay or aberrant colonization may interfere with the development and maturation of the child’s immune system, and thus play a role in the development of allergy and disease [81,82]. There is also accumulating evidence that environmental exposure during the prenatal period can modify gene expression and susceptibility to allergic diseases through epigenetic modifications [83]. It has been reported recently that antibiotic use early in life is associated with the risk of childhood asthma (with a NNTH of 87), allergy, atopic dermatitis, eosinophilic esophagitis, neonatal candidiasis, and celiac disease [84,85,86,87,88,89,90,91,92,93].

Antibiotic overuse during pregnancy is associated with the emergence of many antibiotic-resistant organisms. Rates of GBS resistant to erythromycin (one of the antibiotics of choice after preterm PROM and given in most UK hospitals for that indication) is as high as 35% [94,95]. Their use during pregnancy has been shown also to be associated with the selection of resistant strains of *Escherichia coli*, which has been reported increasingly in neonatal sepsis, especially in very preterm infants [73,96,97].

Some of these risks are inherent to the existing policies for the prevention of pregnancy complications. The policy for the routine administration of antibiotics during cesarean section could be questioned as the risk for postoperative maternal infection varies widely. Among low-risk women (elective cesarean section with intact membranes), approximately 1000 women should receive antibiotics in order to prevent 6 cases of endometritis and 4.4 cases of abdominal wound infections [62,63,98]. In women at higher risk (emergency cesarean section, ruptured membranes, obesity, *etc.*), the number needed to treat is much lower (between 5–25 to avoid one case of maternal postpartum morbidity), but still varies substantially depending on the country [99]. Without further research on long-term infant outcomes, the safest strategy is still to administer antibiotic prophylaxis after fetal cord clamping.

Regarding GBS EOD prophylaxis, rapid GBS detection tests have the potential to detect which mother-infant pairs are at high risk of infection [100,101]. They are rapid and sensitive (85%–98%) enough to obviate the need for prenatal screening. In addition, the new polymerase chain reaction (PCR)-based point-of-care tests can be performed by non-laboratory personnel (*i.e.*, midwives) in the labor suite [102,103,104]. These tests have been shown to be as at least as accurate (sensitivity of 85%) as antenatal culture-based screening (sensitivity of 81%), with the major advantage of identifying those women who are really carrying GBS during labor and thus allow for targeted IAP. Moreover, this approach allows to screen pregnant women whose babies are at higher risk for neonatal sepsis, such as those delivering preterm or not followed during pregnancy [102,103,105]. In my opinion, intrapartum rapid PCR should be the gold standard when deciding to systematically screen GBS in pregnant women. A French group studied the cost of providing PCR in the labor suite for GBS carriage [106]. They showed that the PCR strategy is cost-neutral compared to the antenatal culture strategy if the cost of treating GBS-infected newborns is taken in account, and with the additional benefit of decreasing the incidence of GBS sepsis. However, this technique does not preclude the fact that around 20%–30% of fetuses will receive antibiotics prior to delivery. If our objectives are to prevent GBS early neonatal sepsis as well as *in utero* antibiotic administration, then the solution will be to develop a GBS preventive vaccine.

In women with preterm premature rupture of membranes, co-amoxiclav, a frequently prescribed antibiotic in this context, has been surprisingly associated with an increased risk of necrotizing enterocolitis [28]. In the ORACLE I study, newborns exposed to co-amoxiclav had a 4-fold risk of suspected of proven necrotizing enterocolitis compared to placebo (4.1% *vs.* 2.7; *p* = 0.08) [27]. Co-amoxiclav is known to select for *Clostridium difficile* (a cause of pseudomembranous colitis) in adults. One suggested mechanism of the pathogenesis of neonatal necrotising enterocolitis is the abnormal microbial colonization of the intestinal tract by one or a few species unhindered by competitors. Co-amoxiclav could facilitate such colonization due to its range of activity and effectiveness. Furthermore, the immature gut could absorb the exotoxin produced intact, thus resulting in mucosal damage [107]. Therefore, co-amoxiclav should not be administered in the context of preterm delivery. 

While accepting some elements of risk associated with strong evidence-based preventive policies, the risk of complications associated with antibiotic misuse is not acceptable, e.g., preterm labor with intact membranes, bacterial (including GBS) vaginal colonization during pregnancy, non-specific vaginitis (often of non-infectious origin or “irritative”), or suspected urinary tract infection. In a long-term, follow-up study of infants included in the ORACLE II study (antibiotics *vs.* placebo in women with preterm labor), the risk of cerebral palsy was higher in the group of infants exposed to antibiotics (erythromycin: 3.3% *vs.* 1.7%; OR: 1.93; 95% CI = 1.21–3.09; co-amoxiclav: 3.2% *vs.* 1.9%; OR: 1.69; 95% CI = 1.07–2.67) [26]. The NNTH with erythromycin was 64 (95% CI = 37–209) and 79 with co-amoxiclav (95% CI = 42–591). The authors suggested several possible pathways associated with this association. (1) Antibiotics could help to overcome the infectious insult associated with the preterm labor episode and thus prolong pregnancy, but not necessarily resolving the associated intrauterine and fetal inflammation. A continuing inflammatory environment could have led to fetal brain injury and thereby cerebral palsy. (2) Cerebral palsy could have resulted from a direct effect of the antibiotics themselves. (3) The preterm labor was not associated with infection, but rather with other pathologies within the so-called preterm parturition syndrome, which might have been exacerbated by the antibiotics through mechanisms as yet undetermined. Furthermore, exposure to any macrolide antibiotics (including erythromycin alone or erythromycin plus co-amoxiclav) *vs.* no macrolide antibiotics (including placebo and co-amoxiclav alone) was shown to increase neonatal death (RR: 1.52; 95% CI = 1.05–2.19; NNTH: 139; 95% CI = 61–1429) and any functional impairment (RR: 1.11; 95% CI = 1.01–1.20; NNTH: 24; 95% CI = 13–263). Exposure to any beta-lactam (beta-lactam alone or in combination with macrolide antibiotics) *vs.* no beta-lactam antibiotics resulted also in more neonatal deaths (RR: 1.51; 95% CI = 1.06–2.15; NNTH: 143; 95% CI = 63–1250) [25]. Unfortunately, regardless of the lack of effectiveness and the neonatal and infant long-term risks, antibiotics are still prescribed in the context of preterm labor with intact membranes.

There are also associated risks with the policies of screening and treating of genital infections, such as *T. vaginalis* or BV, for the prevention of preterm birth. In the case of *T. vaginalis*, it has been shown that treatment with metronidazole increased the risk of preterm delivery (19% in the metronidazole group *vs.* 10.7% in the placebo group (RR: 1.8; 95% CI = 1.2–2.7; *p* = 0.004) [108]. It has been hypothesized that the antibiotic induces the liberation of degradation particles, which enhances the inflammatory cascade and therefore preterm labor and delivery. Another problem when diagnosing BV or vaginitis is the use of culture from vaginal secretions. It is well known that cultures should not be used for diagnosing BV and uncomplicated vaginitis. Regrettably, the use of wet mount microscopy is declining and gynecologists often rely on vaginal culture [109,110,111]. In this case, the probability to have a positive vaginal culture is very high (20% for GBS, 10% for *E. coli*, 30%–50% for *Gardenerella vaginalis*, *etc.*), which does not necessarily mean the presence of a pathogenic infection. The end point is that a large number of women receive antibiotics in order to treat their “normal” vaginal flora.

As with genital infections, another problem in obstetrics is the diagnosis of urinary tract infection [31,112]. It is standard of care to perform urinary sticks at each pregnancy visit to search for proteinuria. This allows also to detect the presence/absence of leucocytes in the urine. Approximately 60% of pregnant women have leucocytes in the urinary sticks in the absence of any infectious complication and their presence should not be used for the screening or diagnosis of asymptomatic urinary tract infection. Unfortunately, some clinicians prescribe antibiotics for a suspicion of urinary tract infection or perform urinary culture. When treating the women with antibiotics due to the presence of leucocytes in the sticks, they are giving them for no valid reason and exposing the woman and fetus to potential side-effects. When performing culture, they are increasing healthcare costs as most results will come back as “mixed flora” or contamination.

As mentioned previously, intrapartum choriamnionitis should be treated promptly with antibiotics to prevent at least maternal complications. Fever is one of the symptoms that may be present with choriamnionitis [113]. However, the most frequent reason for fever during labor and delivery is not choriamnionitis, but prolonged labor (per each additional hour: OR: 1.15; 95% CI = 1.08–1.22) or epidural analgesia (11.8% of women) [114,115,116]. Therefore, a complete check-up, including maternal and fetal signs and symptoms, as well as a blood examination including white cell counts and CRP, should be performed before starting intravenous antibiotics [113]. Consultation with obstetric providers to determine whether chorioamnionitis is suspected is important for guiding neonatal management. If antibiotics have been prescribed, their duration should be as short as possible in order to minimize exposure to the newborn through breast milk [117]. Several studies have shown that the administration of just one dose of antibiotics after delivery is equally effective than a longer duration of treatment [118,119].

## 4. Conclusions

The good intentions of obstetricians to reduce maternal and fetal/neonatal infectious complications are unfortunately impacting on the microbial milieu for the next generation. The growing concern related to the short- and long-term consequences of antibiotic administration requires better diagnostic strategies, as well as new strategies for the risk stratification of pregnant women. For every pregnant woman, we should weigh the risks and benefits of antibiotics for both her and her fetus/newborn/infant and encourage patients to be part of a shared decision-making process. In addition, recommendations should be discussed and adopted within the local context. For example, GBS prophylaxis or antibiotics prior to cesarean section may be recommended in some countries, such as the USA, but may not be applicable in some European countries with lower infection rates. The recent resolution of the 2014 World Health Assembly has clearly sounded the alarm by recognizing that antibiotic resistance through the abuse and misuse of these drugs is a major public health issue and “urges member states to strengthen drug management systems…and to encourage the development of new diagnostics and treatment options”. Let us hope that this message has been well understood by those in the field of obstetrics.
